# Association Between Dietary Habits in Midlife With Dementia Incidence Over a 20-Year Period

**DOI:** 10.1212/WNL.0000000000201336

**Published:** 2023-01-03

**Authors:** Isabelle Glans, Emily Sonestedt, Katarina Nägga, Anna-Märta Gustavsson, Esther González-Padilla, Yan Borne, Erik Stomrud, Olle Melander, Peter M. Nilsson, Sebastian Palmqvist, Oskar Hansson

**Affiliations:** From the Clinical Memory Research Unit (I.G., K.N., A.-M.G., Erik Stomrud, S.P., O.H.), Department of Clinical Sciences Malmö, Lund University, Sweden; Memory Clinic (I.G., A.-M.G., Erik Stomrud, S.P., O.H.), Skåne University Hospital, Malmö, Sweden; Department of Clinical Sciences in Malmö(Emily Sonestedt, E.G.-P., Y.B.), Nutritional Epidemiology, Lund University, Skåne University Hospital, Malmö, Sweden; Department of Acute Internal Medicine and Geriatrics (K.N.), Linköping University, Sweden; and Department of Clinical Sciences Malmö (O.M., P.M.N.), Lund University, Skåne University Hospital, Malmö, Sweden.

## Abstract

**Background and Objectives:**

Dementia cases are expected to triple during the next 30 years, highlighting the importance of finding modifiable risk factors for dementia. The aim of this study was to investigate whether adherence to conventional dietary recommendations or to a modified Mediterranean diet are associated with a subsequent lower risk of developing all-cause dementia, Alzheimer disease (AD), vascular dementia (VaD), or with future accumulation of AD-related β-amyloid (Aβ) pathology.

**Methods:**

Baseline examination in the prospective Swedish population-based Malmö Diet and Cancer Study took place in 1991–1996 with a follow-up for incident dementia until 2014. Nondemented individuals born 1923–1950 and living in Malmö were invited to participate. Thirty thousand four hundred forty-six were recruited (41% of all eligible). Twenty-eight thousand twenty-five had dietary data and were included in this study. Dietary habits were assessed with a 7-day food diary, detailed food frequency questionnaire, and 1-hour interview. Main outcomes were incident all-cause dementia, AD, or VaD determined by memory clinic physicians. Secondary outcome was Aβ-accumulation measured using CSF Aβ42 (n = 738). Cox proportional hazard models were used to examine associations between diet and risk of developing dementia (adjusted for demographics, comorbidities, smoking, physical activity, and alcohol).

**Results:**

Sixty-one percent were women, and the mean (SD) age was 58.1 (7.6) years. One thousand nine hundred forty-three (6.9%) were diagnosed with dementia (median follow-up, 19.8 years). Individuals adhering to conventional dietary recommendations did not have lower risk of developing all-cause dementia (hazard ratio [HR] comparing worst with best adherence, 0.93, 95% CI 0.81–1.08), AD (HR 1.03, 0.85–1.23), or VaD (HR 0.93, 0.69–1.26). Neither did adherence to the modified Mediterranean diet lower the risk of developing all-cause dementia (HR 0.93 0.75–1.15), AD (HR 0.90, 0.68–1.19), or VaD (HR 1.00, 0.65–1.55). The results were similar when excluding participants developing dementia within 5 years or those with diabetes. No significant associations were found between diet and abnormal Aβ accumulation, conventional recommendations (OR 1.28, 0.74–2.24) or modified Mediterranean diet (OR 0.85, 0.39–1.84).

**Discussion:**

In this 20-year follow-up study, neither adherence to conventional dietary recommendations nor to modified Mediterranean diet were significantly associated with subsequent reduced risk for developing all-cause dementia, AD dementia, VaD, or AD pathology.

The estimated number of dementia cases was globally 47 million in 2015 and is expected to triple during the next 30 years.^1^ Because effective treatment is lacking, effectively targeting modifiable risk factors for cognitive impairment and dementia could provide great benefits for this population and reduce societal costs. In addition to a large burden for patients and relatives, there is a tremendous burden for the health care system, with a global cost of US$1 trillion annually.^2^ As acknowledged by the 2020 report of the Lancet Commission on Dementia prevention, intervention, and care, modifiable risk factors account for 40% of worldwide dementia cases.^2^

A modifiable and controversial risk factor for cognitive impairment and dementia is dietary habits. Several studies have examined how dietary habits affect incidence in dementia disorders, with inconsistent results. Systematic reviews and meta-analyses conclude that adhering to Mediterranean diet may contribute to a slowing of cognitive decline and a lower incidence in dementia.^3-5^ However, there are several important methodological weaknesses in many of the previous studies, including (1) exclusively relying on data from retrospective food frequency questionnaires (FFQs) with possible report biases; (2) insufficient follow-up time; (3) inclusion of participants older than 70 years, with a possible cognitive impairment already affecting diet (i.e., reversed causality); and (4) usage of all-cause dementia as outcome, missing the possibility of diet being differently associated with specific dementia diseases, such as Alzheimer disease (AD), which is the most common accounting for 60%–70% of all dementia cases, and the second most common caused by cerebrovascular disease vascular dementia (VaD), which exhibit different genetic and lifestyle risk factor patterns.

To further understand the mechanisms between a potential dietary influence on the incidence of specific dementia disorders, it would be an advantage to examine the association between diet and the underlying disease pathology. Accumulation of amyloid-β (Aβ) in the brain is the cause of AD, and it can be detected using CSF analysis of Aβ42 or Aβ PET imaging.^1^ However, large-scale longitudinal studies evaluating potential associations between midlife diet and amyloid pathology are lacking.

In this observational study, we prospectively collected detailed dietary data in midlife from a large population-based study of more than 28,000 individuals. The aim was to examine the association between adherence to general dietary guidelines and Mediterranean diet of dementia incidence. Development of any kind of dementia during 20 years was used as primary outcome. Secondary outcomes were development to specifically AD dementia or VaD, respectively. In a convenience subsample (n = 738), we performed an exploratory analysis studying the association between dietary components and future accumulation of AD-related pathology measured using CSF analysis of Aβ42.

## Methods

### Standard Protocol Approvals, Registrations, and Patient Consents

All participants received information about the study and gave written consent to participate. Ethical approval was given by the Ethical Committee of Lund University, Lund, Sweden (LU 90-51).

### Study Population

Data were obtained from the Malmö Diet and Cancer study (MDCS), which is a population-based prospective cohort study from the city of Malmö, Sweden. Between 1991 and 1996, men born in 1923–1945 and women born 1923–1950 living in Malmö were deemed eligible and invited to participate. Exclusion criteria were language problems and mental incapacity, which could preclude participants from fulfilling the questionnaires properly. In all, 30,446 individuals attended at least a part of the baseline examination, which included a self-administrated questionnaire that assessed education, occupation, physical activity, social network, use of tobacco and alcohol, current health, medical history, current medication, and family history of disease in close relatives, along with body composition measurements and dietary assessments. Participation reached 40.8% of the eligible population.^6^

A subpopulation of the study participants underwent lumbar puncture with analyses of CSF Aβ42 when referred to the Memory clinic at Skåne University Hospital, Malmö, after developing clinical signs of cognitive impairment. This represents a convenience subsample of the MDCS and should not be regarded as a representative subsample of the whole study.

### Dietary Assessments and Method

Information on dietary habits was obtained from all participants at baseline through (1) a diet diary and (2) a self-administered FFQ, which provided complementary information, and (3) a 45–60 minute interview with trained personnel.^7^ Detailed information on the dietary data collection procedure, including administration of the FFQ and diary, was given at the first visit by trained research nurses. In the 7-day food diary, participants recorded cooked meals, cold beverages, drugs, natural remedies, and dietary supplements. In the 168-item FFQ participants stated, with reference period last year, regularly consumed foods not covered by the food diary (i.e., breakfast and snacks), portion size (based on pictures of 4 portion sizes for 48 food items), and frequency of food consumed. In addition, participants underwent a 1-hour interview with trained personnel collecting additional information on cooking methods, food choices, and portion sizes and to make sure the information from the diary and FFQ was correct. Halfway through the baseline data collection (in 1994), the interview routines changed to reduce interview time to 45 minutes.^8^ Average daily food intake (g/d) was calculated by combining the data from the food diary and the FFQ. Energy and nutrient intakes were calculated by combining the data on average food intake with the MDCS database originating from the Swedish National Food agency database. The combined dietary method used in MDCS has been validated against 18 days of weighted food records with correlation coefficients for men/women of 0.60/0.74 for sugar, 0.74/0.69 for fiber, 0.65/0.58 for vegetables, 0.60/0.77 for fruits, 0.35/0.65 for fish, 0.82/0.91 for meat, and 0.50/0.65 for wine.^7,9^

### Swedish Dietary Guidelines Score and Modified Mediterranean Diet Score

A Swedish dietary guidelines score (SDGS), designed to portray a healthy dietary pattern, based on Swedish nutrition recommendations and dietary guidelines,^10^ which are in line with dietary recommendations in the United States^11^ and United Kingdom,^12^ was calculated for each participant based on average daily food intake. The SDGS is based on consumption of 5 dietary domains: dietary fiber ≥2.4 g/MJ from nonalcohol energy; added sugar ≤10% of nonalcohol energy; fish and shellfish ≥300 g/wk; fruit and vegetables ≥400 g/d; and red and processed meat ≤500 g/wk. Reaching recommendations yielded 1 point/domain, with a maximum SDGS of 5 points. A similar index has previously been developed within MDCS.^13^ In the modified index, we included added sugar (instead of sucrose) and red and processed meat (instead of saturated and polyunsaturated fat). Based on the SDGS, participants were divided into the following groups: low adherence (0–1 points), moderate adherence (2–3 points), and high adherence (4–5 points).

A modified Mediterranean diet score (mMDS) was created based on the original 14-item score used for the Prevención con Dieta Mediterránea (PREDIMED) study.^14^ The mMDS consists of 10 dietary domains: (1) Vegetable oils (≥54 g/d); (2) Vegetables including legumes (≥300 g/d); (3) Fruits (≥300 g/d); 4) Red and processed meat (<125 g/d); (5) Butter, margarine, cream (<12 g/d); (6) Soda (<200 g/d); (7) Wine (≥100 g/d); (8) Fish and seafood (≥53.6 or ≥85.7 g/d, respectively); (9) Pastries and candy (<21.4 or <12.8 g/d, respectively); and (10) Nuts and seeds (≥12.8 g/d). Reaching recommendations yielded 1 point/domain with a maximum mMDS of 10 points (meets all recommendations). Domain “legumes” was incorporated in the vegetable domain. In addition, “sofrito,” a typical Mediterranean sauce, was excluded in the mMDS because of low consumption in Nordic countries and the lack of information from the food records. Two domains were further excluded from the index (olive oil as main culinary lipid and poultry more than red meats) because of lack of questions referring to this in the present FFQ.^14^ However, the consumption of olive oil was added to the intake of other vegetable oils because the consumption of these oils is usually low in the Nordic population. Based on the mMDS, participants were divided into the following groups: low adherence (0–1 points), moderate adherence (2–4 points), and high adherence (5–10 points). The regrouping was performed to have enough discrepancies between groups regarding dietary habits and to avoid too small groups (eTable 1, links.lww.com/WNL/C345).

A Mediterranean diet score according to a study^15^ was calculated scoring 0–50 (poor to good adherence to Mediterranean diet recommendations). The original score is from 0 to 55, but here, the domain legumes was incorporated in the domain “vegetables” because of low consumption in Nordic population. As in the case of mMDS, all vegetable oils were considered rather than olive oil alone (eTable 2, links.lww.com/WNL/C345).

### Covariates

Sociodemographic factors included age, sex, and education. At baseline, participants completed a questionnaire including lifestyle factors and health status. Education level was divided into 3 subgroups as per study design: primary/elementary school (≤8 years), secondary school/high school (9–12 years), or higher education/university (≥13 years), based on information from the questionnaire. From the questionnaire smoking status was divided into 3 groups (smokers, former smokers, and never smokers) and physical activity as metabolic equivalent hours/week (METh/week). One METh is defined as the metabolic intensity when a person is at rest. METh/week was computed by multiplying time (hours) spent on each activity by the respective MET (intensity) factor.^2^ Information on alcohol consumption was derived both from the questionnaire and the 7-day record. Zero consumers had reported no consumption during the past year. The other participants were stratified in quintiles separately in men and women (because of alcohol consumption and daily recommendations differ between men and women), with the following spans: 0–3.4, 3.4–9.1, 15.7, and 25.7 g/d (men) and 0–0.9, 4.3, 8.1, and 14.0 g/d (women). *APOE*-ɛ4 interaction analysis was performed to investigate a potentiating effect of this well-established genetic risk factor for AD. Owing to possible seasonal variations in dietary intake, and that dietary interview was shortened in 1994, season and diet assessment method were included in the analyses.

### Outcomes

The primary outcome was progression to all-cause dementia disorders. The secondary outcomes were progression to AD dementia and VaD. The exploratory outcome was abnormal Aβ status measured as increased levels of CSF Aβ42 (see *Exploratory outcome CSF Aβ42* below).

The primary and secondary diagnostic outcomes were based on registered dementia diagnoses from the Swedish National Patient Register (NPR) throughout 2014. The NPR covers both the Swedish Inpatient Register and the hospital-based outpatient register. Diagnoses included AD dementia (*ICD-10* and *ICD-9* codes F00, G30, 331A/331.0), VaD (F01, 290E/290.4), Parkinson disease dementia (F023), dementia with Lewy bodies (F028, G318A), frontotemporal dementia (F020, G310, 331B/331.1), or unspecified dementia (F03, 290, 294B/294.1, 331C/331.2). After the register data delivery, trained physicians with special interest in dementias, but not yet board-certified specialists, at the Memory Clinic at Skåne University Hospital reviewed and validated all registered dementia diagnoses based on symptoms, results of cognitive tests, brain imaging (CT or magnetic resonance imaging reviewed by radiologists), and CSF concentrations of Aβ42 and tau phosphorylated at Thr181 (p-tau, when available) in accordance with the specific major neurocognitive disorders in *DSM-5* (*Diagnostic and Statistical Manual of Mental Disorders, Fifth Edition*). In uncertain cases, 2 specialists in Neurology and Geriatrics, (O.H. and K.N.), respectively, with more than 10 years of experience in the field of dementia were involved. In cases where CSF was available, AD diagnosis was based on the NIA-AA criteria.^16^ Of the 1,943 cases with dementia, 1,507 (77.6%) were diagnosed at a specialized Memory Clinic.

The secondary outcomes were defined as pure AD, mixed AD and vascular pathology, and VaD. Information from neuroimaging was used to validate VaD and to differentiate between pure AD and AD with cerebrovascular disease (mixed AD and vascular pathology). Presence of significant cerebrovascular disease on neuroimaging led to a mixed AD diagnosis if AD was considered the primary cause.

### Exploratory Outcome—CSF Aβ42

CSF data were available for participants with signs of cognitive decline who had been referred to the Memory Clinic at Skåne University Hospital for further investigation. With lumbar puncture, CSF was collected between 1995 and 2015 according to a structured protocol.^19^ CSF concentrations of Aβ42 were measured using INNOTEST ELISA (Fujirebio Europe, Ghent, Belgium).

Cut-off values for CSF Aβ42 were established using mixture modeling.^17-22^ Because of a slight, assay-dependent drift in levels of CSF Aβ42 during the collection period 1995–2015, 2 different cutoffs were established for the period 1995–2003 (Aβ42 < 484.8 pg/mL) and 2004–2015 (Aβ42 < 577.1 pg/mL). This drift in INNOTEST CSF Aβ42 values during this period is well known.^19^

### Statistical Analyses

Demographic data are presented as means (SDs) or numbers (n) and percent (%). Cox proportional hazard models, with years between baseline and event as time variable, were used to examine associations between SDGS and mMDS and risk of developing dementia. Event was defined as *all-cause dementia* (primary outcome) or *specific dementia disorder* (secondary outcome). Censoring occurred at the recorded date of dementia, time of death, or at time of register data delivery (December 31, 2014). By using this approach, the competing risk of death was assessed by estimating cause-specific hazard ratio (HR) for dementia, in accordance with recommendations when the study objective is etiologic.^23^ All analyses were adjusted for age, sex, education, season, dietary sampling method, and total energy intake (kJ) in model 1. In model *2*, the following lifestyle variables were also included: smoking (current, former, never), alcohol consumption (sex-specific quintiles), body mass index, and physical activity (METh/week). In model *2*, a total of 243 cases had missing data in any of these lifestyle variables and were thus excluded. Logistic regression analysis was used to examine the association between SDGS and Aβ42 accumulation, using CSF Aβ42 status as outcome.

In sensitivity analyses, participants diagnosed with dementia <5 years from baseline were excluded, to reduce the possible bias of cognitive impairment affecting dietary intake^24^ (i.e., a preclinical or prodromal dementia disorder). A total of 73 were excluded in this analysis. In another sensitivity analysis, participants with prevalent and incident diabetes mellitus were excluded to investigate the dietary association without being influenced by diabetes and the dietary restrictions that may accompany that disease.^25^ A total of 5,221 participants with diabetes were excluded; of which, 405 developed dementia. Sensitivity analysis was also performed excluding participants that had made substantial changes in their dietary habits at any time before the baseline examination. In addition, another sensitivity analysis was performed only including individuals diagnosed at the Memory Clinic. Using the Mediterranean diet score according to a study^15^ was used in another sensitivity analysis.

All statistical analyses were performed using R (version 3.6.3). A *p*-value <0.05 was considered significant.

### Data Availability

The data that support the findings of this study are available from the Malmö Population-Based Cohorts Joint Database, but restrictions apply to the availability of these data, which were used under license for the current study, and so are not publicly available. Data are however available from the authors on reasonable request and with permission of the Malmö Population-Based Cohorts Joint Database.

## Results

### Baseline Characteristics

**Figure 1:**
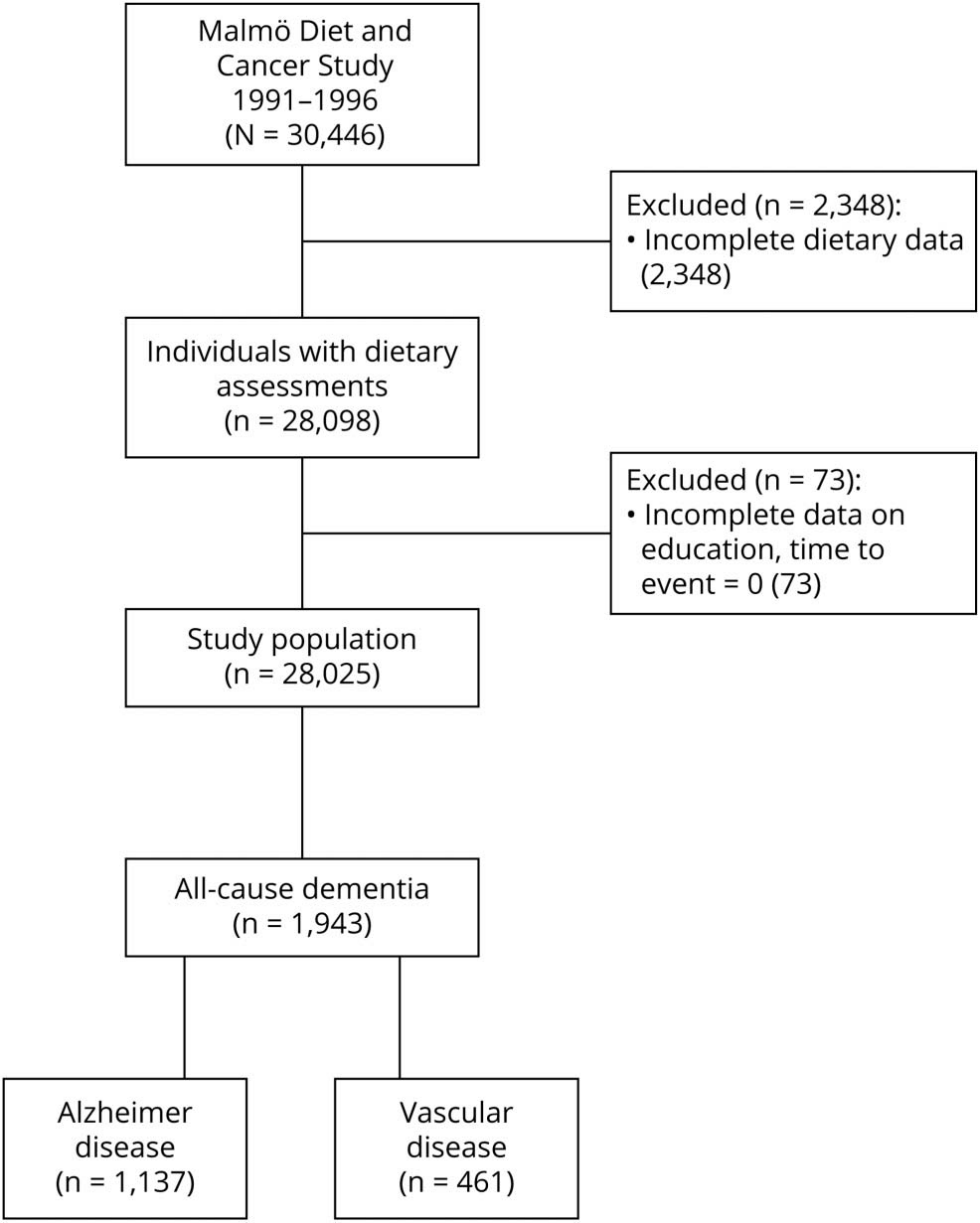
Flowchart Describing the MDCS Population at Baseline and Follow-up

Individuals who underwent dietary assessments were included (n = 28,098) in this study. Participants with incomplete data on education (n = 71) and where time to event was zero, meaning present dementia (n = 2), were excluded, resulting in a complete data set of 28,025 individuals, which was used for the main analysis. See Figure 1 for an enrollment flowchart. Of the 28,025 dementia-free participants at study recruitment in 1991–1994, a total of 1,943 (6.9%) were diagnosed with dementia during a median of 19.8 (interquartile range [IQR] 4.8) years of follow-up. Comparison of baseline characteristics between complete data set and excluded group is shown in eTable 3 (links.lww.com/WNL/C345). Table 1 presents characteristics of participants who subsequently developed any type of dementia (all-cause dementia) compared with those who did not develop dementia. The individuals who subsequently developed dementia were significantly older at baseline and had a lower level of the education. There was no difference in sex, 61% were women in both groups. Neither SDGS nor mMDS differed between groups. Median time from baseline to any dementia diagnosis was 15.5 (IQR 6.7) years. Baseline characteristics across dietary adherence levels are shown in eTables 4–5 (links.lww.com/WNL/C345).

**Table 1:**
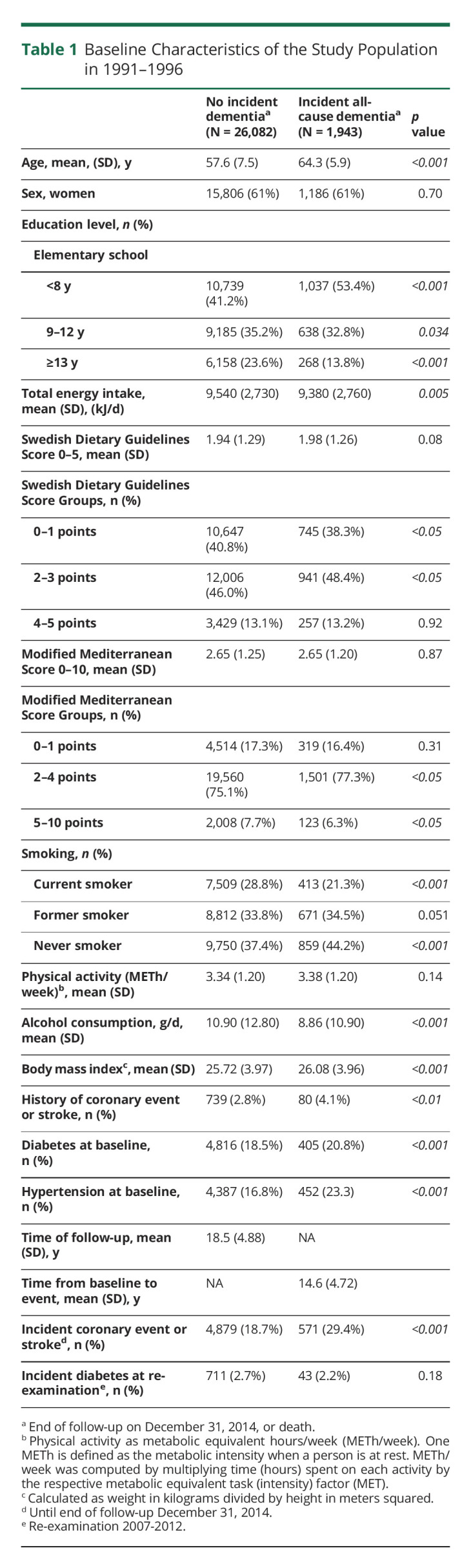
Baseline Characteristics of the Study Population in 1991–1996

### Association Between Diet and Risk of Developing All-Cause Dementia

Associations between SDGS and development of all-cause dementia, AD dementia, and VaD are presented in Table 2. Individuals who adhered to the dietary recommendations did not show a significantly lower risk of developing all-cause dementia in model 1 (HR 0.98, 95% CI: 0.95–1.02) or model 2 (HR 0.99, 95% CI: 0.95–1.03) using SDGS as a continuous variable (0–5, poor to good adherence). Furthermore, individuals adhering to dietary recommendations (SDGS 4–5) did not have lower risk of developing dementia in either model 1 (HR = 0.93; 95% CI: 0.81–1.08) nor in model 2 (HR 0.95; 95% CI: 0.82–1.10) compared with those with low (SDGS 0–1), Table 2.

**Table 2:**
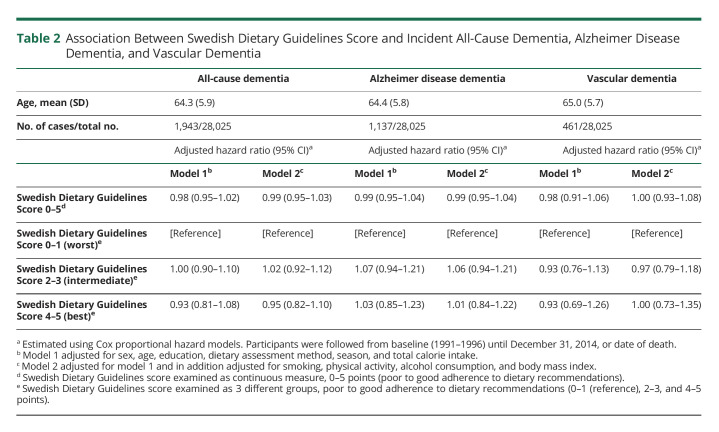
Association Between Swedish Dietary Guidelines Score and Incident All-Cause Dementia, Alzheimer Disease Dementia, and Vascular Dementia

Neither did adherence to the modified Mediterranean diet lower the risk of developing all-cause dementia in model 1 (HR 0.93, 95% CI: 0.75–1.15) or model 2 (HR 1.00, 95% CI: 0.96–1.04) when using mMDS as a continuous variable. Individuals adhering to the modified Mediterranean diet (mMDS 5–10) did not have lower risk of developing all-cause dementia (HR 0.93, 95% CI 0.75–1.15) in model 1 and (HR 0.95 95% CI: 0.76–1.18) in model 2 (Table 3).

**Table 3:**
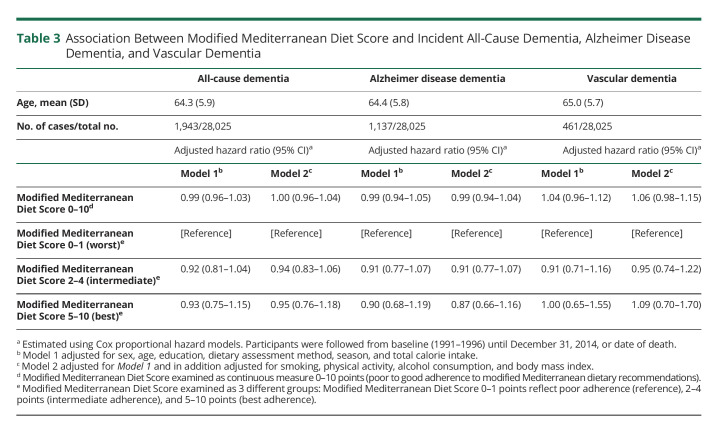
Association Between Modified Mediterranean Diet Score and Incident All-Cause Dementia, Alzheimer Disease Dementia, and Vascular Dementia

### Association Between Diet and Risk of Developing Alzheimer Disease or VaD

As shown in Table 2, no significant associations between SDGS as continuous variable and development of AD dementia (HR 0.99, 95% CI: 0.95–1.04) or VaD (HR 0.98, 95% CI: 0.91–1.06) were found. Individuals adhering to dietary recommendations (SDGS 4–5) did not have lower risk of developing AD dementia (HR 1.03, 95% CI: 0.85–1.23) or VaD (HR 0.93, 95% CI: 0.69–1.26) compared with those with low SDGS (0–1), AD dementia (HR 0.90, 95% CI: 0.68–1.19), or VaD (HR 1.00, 95% (CI: 0.65–1.55) (Table 2).

Neither did adherence to modified Mediterranean diet lower the risk of developing AD dementia (HR 0.99 95% CI: 0.94–1.05) or VaD (HR 1.04 95% CI: 0.96–1.12) when analyzing mMDS as a continuous variable. Individuals with the highest adherence to the modified Mediterranean diet (mMDS 5–10) did not have lower risk of developing AD dementia (HR 0.90 95% CI: 0.68–1.19) or VaD (HR 1.00 95% CI: 0.65–1.55) (Table 3). Adjusted Kaplan-Meier curves displaying no effects of diet quality and incidence in all-cause dementia, ADD, or VaD are shown in eFigure 1 (links.lww.com/WNL/C345).

### Sensitivity Analyses

In sensitivity analyses, we excluded participants with incident dementia diagnosis within 5 years after baseline and participants with prevalent or incident diabetes, respectively. As for the main results, no significant effect of lowering the risk of dementia was found between SDGS or the mMDS with development of all-cause dementia, AD dementia, or VaD (eTables 6–9, links.lww.com/WNL/C345).

Furthermore, in sensitivity analyses excluding participants indicating a substantial change in their dietary habits before baseline, no significant effect of lowering the risk of dementia was found between SDGS or the mMDS with development of all-cause dementia, AD dementia, or VaD (eTables 10–11, links.lww.com/WNL/C345).

In sensitivity analysis using the Mediterranean diet score according to a study,^15^ no significant effect of lowering the risk of all-cause dementia, AD dementia, or VaD was found (eTable 12, links.lww.com/WNL/C345).

Furthermore, there were no significant interaction effects between SDGS or the mMDS and the AD risk genotype *APOE* ε4 (eTables 13–14, links.lww.com/WNL/C345).

Excluding participants not diagnosed at a Memory Clinic did not change the results (data not shown).

### Diet and Deposition of CSF β-Amyloid

A total of 777 participants underwent lumbar puncture. Participants with missing CSF Aβ42 data were excluded (n = 39), resulting in a sample of 738 participants with available CSF Aβ42 data. Median time from baseline to CSF collection was 12.9 (IQR 7.4) years. Associations between SDGS and CSF Aβ42 status (normal/abnormal) are shown in the eTable 15 (links.lww.com/WNL/C345). No significant association between SDGS and CSF Aβ42 at follow-up was found. Individuals with the highest adherence to dietary recommendations (SDGS 4–5) did not have lower risk of having abnormal Aβ42 in CSF (OR 1.10, 95% CI: 0.66–1.84).

Associations between mMDS and CSF Aβ42 status (normal/abnormal) are shown in eTable 16 (links.lww.com/WNL/C345). No significant associations between mMDS and CSF Aβ42 at follow-up were found. Individuals with the highest adherence to the mMDS (5–10) did not have lower risk of having abnormal Aβ42 in CSF (OR 0.82, 95% CI: 0.37–1.79; *p* = 0.69). In addition, adjusting for time between baseline and CSF collection did not change the results. No significant association was found between Mediterranean diet score measured according to another study,^15^ and abnormal Aβ42 in CSF (eTable 17, links.lww.com/WNL/C345).

## Discussion

Midlife self-reported diet quality according to dietary recommendations or according to the modified Mediterranean diet were not associated with a subsequent reduced risk of developing all-cause dementia, AD dementia, or VaD over a median follow-up time of 19.8 years in this population-based prospective cohort study of 28,025 individuals. Furthermore, diet was not associated with presence of Aβ pathology at follow-up.

The main strengths of this study were the prospective study design, the large sample size, the long follow-up time of almost 20 years, and the dietary assessment method of high quality, where FFQ was complemented by interviews, which improves the validity of the dietary data.^13^ In addition, the dementia diagnoses were validated by trained physicians at a Memory Clinic and are not merely register-based as in several previous publications.^26^ Another strength is the mMDS specifically created for the MDCS based on the Med Diet Score used for the PREDIMED study, which has been validated as an accurate measurement of adherence to Mediterranean diet.^14^

Previous studies on diet quality and incidence of dementia disorders have shown varying results. In a large prospective cohort study (n = 10,308), with a median follow-up time of 24.8 years, neither the quality of midlife diet nor the Mediterranean diet was associated with incident all-cause dementia,^26^ which was confirmed by another prospective and longitudinal study with 20 years of follow-up including 13,588 participants showing no association of diet quality and further development of dementia or cognitive decline.^27^ Another study^28^ found no associations between the Mediterranean diet and cognitive decline or dementia; they could however show an association between MIND (Mediterranean-DASH Intervention for Neurological Delay) diet and cognitive decline as well as dementia, in 1,220 participants over a 12-year period. A systematic review^3^ including 32 studies, concluded that adherence to a Mediterranean diet may contribute to better cognitive performance and decreased risk of developing dementia. This review reflects the current state of knowledge because the studies included are very heterogeneous in their designs. A majority of them used retrospective FFQs, which may be associated with misreporting by the participants considering difficulties to remember historical details about food intake many years or even decades back in time.^29^ In addition, a majority of the studies included in the review are based on dietary habits in older populations (older than 65 years). Considering the relatively high prevalence of prodromal dementias in such populations, there is a risk of reversed causality. Early cognitive decline with possible mood changes could also affect dietary lifestyle habits.^30^ Hence, inclusion of an older population may bias the correlation between diet quality and cognitive decline. In addition, the varying follow-up time between studies might be one reason of the heterogeneous study results regarding dietary habits and incidence of dementia disorders. However, the authors of the systematic review concluded and emphasized the importance of confirmation based on large longitudinal epidemiologic studies with prospective registration of dietary habits, thus highlighting the value of this study. As expected, known cardiovascular risk factors such as coronary event or stroke, diabetes, and arterial hypertension were all more common at baseline in the group with incident dementia (Table 1).

This study does not exclude a possible association between diet quality and subsequent development of dementia. However, the present Swedish dietary recommendations, which are in line with those in the United Kingdom and United States, or according to the Mediterranean dietary pattern, could not be confirmed to be associated with prevention of dementia. There is however a risk of self-reported dietary and lifestyle habits being misreported to some extent. There is also a possible risk of alteration in dietary habits, although dietary habits remain relatively stable during life. Previous studies on diet and CSF Aβ measures are either cross-sectional^31,32^ or have short follow-up time (4 weeks–1.2 years).^33^ This further highlights the importance of this study examining the association between dietary habits and later CSF Aβ-pathology, with 13 years of follow-up. However, 1 review, including 7 RCTs, 7 cross-sectional studies, and 1 longitudinal study on diet and biomarkers of AD, concludes that adherence to Mediterranean diet and reduction of AD biomarkers, as well as high-glycemic and high saturated fat diet, were all associated with an increase in biomarker levels of AD.^34^ A study showed that adherence to the Mediterranean diet was associated with better cognitive performance in midlife.^35^ However, we emphasize using dementia diagnoses as outcome is the most clinically relevant.

Considering that no systematic cognitive testing was available during follow-up (only available in medical records for those undergoing dementia workups according to routine clinical practice), and that the reviewed dementia diagnoses were retrieved from the NPR, there could be an underestimation of the number of dementia cases. The advantage, however, is that all surviving participants were under risk for dementia during the study period and only those completing follow-up visits. Note also that to improve the quality of the dementia diagnoses, fulfillment of diagnostic criteria was validated retrospectively by trained physicians at the memory clinic. Dietary data were only collected at baseline, and there is a risk of alterations in dietary habits during follow-up. This was however somewhat accounted for by excluding participants who reported a substantial alteration in dietary habits at any time before baseline in sensitivity analysis, with the same results as in the main analyses. Although participants were followed for a median period of 19.8 years, we cannot rule out that even longer follow-up might have resulted in a slightly different result. Using vegetable oils instead of olive oil could be a limitation of the mMDS; however, the vegetable oils in the mMDS are all of vegetable origin, with a high proportion of monosaturated and polyunsaturated fatty acids. Furthermore, participants with CSF samples were not randomized but recruited based on clinical indications, which is a limitation of this study. Although multiple baseline characteristics were adjusted for, additional possible confounding factors cannot be ruled out, such as health selection bias. Randomized controlled trials are needed to provide additional evidence regarding the potential role of diet in relation to AD pathology. However, it is probably not feasible to design a 20-year randomized controlled trial with strict dietary habits to adhere to.

Adherence to conventional dietary recommendations or to a modified Mediterranean diet during midlife were not associated with a lower incidence in all-cause dementia over a 20-year period, nor was the diet associated with AD dementia, accumulation of Aβ pathology, or to VaD. Large intervention studies are needed to clearly understand whether diet quality can affect incidence of dementia disorders, even if the results from this study together with other large prospective longitudinal studies^26,27^ implicate this will not be the case.

## Glossary

Aβ: AD-related β-amyloid
AD: Alzheimer disease
FFQ: food frequency questionnaire
mMDS: modified Mediterranean diet score
IQR: interquartile range
METhh/week: metabolic equivalent hours/week
NPR: National Patient Register
PREDIMED: Prevención con Dieta Mediterránea
SDGS: Swedish dietary guidelines score
VaD: vascular dementia

## References

[1] Hansson O. Biomarkers for neurodegenerative diseases. Nat Med. 2021;27(6):954-963. 34083813 10.1038/s41591-021-01382-x

[2] Livingston G, Huntley J, Sommerlad A, et al. Dementia prevention, intervention, and care: 2020 report of the Lancet Commission. Lancet. 2020;396(10248):413-446. 32738937 10.1016/S0140-6736(20)30367-6 PMC7392084

[3] Petersson SD, Philippou E. Mediterranean diet, cognitive function, and dementia: a systematic review of the evidence. Adv Nutr. 2016;7(5):889-904. 27633105 10.3945/an.116.012138 PMC5015034

[4] Yusufov M, Weyandt LL, Piryatinsky I. Alzheimer's disease and diet: a systematic review. Int J Neurosci. 2017;127(2):161-175. 26887612 10.3109/00207454.2016.1155572

[5] Singh B, Parsaik AK, Mielke MM, et al. Association of mediterranean diet with mild cognitive impairment and Alzheimer's disease: a systematic review and meta-analysis. J Alzheimer's Dis. 2014;39(2):271-282. 24164735 10.3233/JAD-130830 PMC3946820

[6] Manjer J, Carlsson S, Elmståhl S, et al. The Malmö diet and cancer study: representativity, cancer incidence and mortality in participants and non-participants. Eur J Cancer Prev. 2001;10(6):489-499. 11916347 10.1097/00008469-200112000-00003

[7] Riboli E, Elmståhl S, Saracci R, Gullberg B, Lindgärde F. The Malmö food study: validity of two dietary assessment methods. Int J Epidemiol. 1997;26:161-173. 10.1093/ije/26.suppl_1.s161 9126544

[8] Wirfält E, Mattisson I, Johansson U, Gullberg B, Wallström P, Berglund G. A methodological report from the Malmö Diet and Cancer study: development and evaluation of altered routines in dietary data processing. Nutr J. 2002;1:3. 12537595 10.1186/1475-2891-1-3 PMC149436

[9] Elmståhl S, Riboli E, Lindgärde F, Gullberg B, Saracci R. The Malmö food Study: the relative validity of a modified diet history method and an extensive food frequency questionnaire for measuring food intake. Eur J Clin Nutr. 1996 Mar;50(3):143-51. 8654327

[10] FAO. Food-Based Dietary Guidelines-Sweden [online]. Updated 2022. Accessed March 17, 2022. https://www.fao.org/nutrition/education/food-based-dietary-guidelines/regions/countries/sweden/en/.

[11] United States Department of Agriculture and the Department of Health and Human Services. Dietary Guidelines for Americans, 2020-2025. 9th ed; updated 2020. Accessed September 16, 2021. pcrm.org/good-nutrition/nutrition-programs-policies/2020-2025-dietary-guidelines.

[12] Public Health England. Government Dietary Recommendations. 2016. Accessed September 16, 2021. assets.publishing.service.gov.uk/government/uploads/system/uploads/attachment_data/file/618167/government_dietary_recommendations.pdf.

[13] Drake I, Gullberg B, Ericson U, et al. Development of a diet quality index assessing adherence to the Swedish nutrition recommendations and dietary guidelines in the Malmö Diet and Cancer cohort. Public Health Nutr. 2011;14(5):835-845. 21299917 10.1017/S1368980010003848

[14] Martinez-Gonzalez MA, Garcia-Arellano A, Toledo E, et al. A 14-item Mediterranean diet assessment tool and obesity indexes among high-risk subjects: the PREDIMED trial. PLoS One. 2012;7(8):e43134. 22905215 10.1371/journal.pone.0043134 PMC3419206

[15] Panagiotakos DB, Pitsavos C, Stefanadis C. Dietary patterns: a Mediterranean diet score and its relation to clinical and biological markers of cardiovascular disease risk. Nutr Metab Cardiovasc Dis. 2006;16(8):559-568. 17126772 10.1016/j.numecd.2005.08.006

[16] Albert MS, DeKosky ST, Dickson D, et al. The diagnosis of mild cognitive impairment due to Alzheimer's disease: recommendations from the National Institute on Aging-Alzheimer's Association workgroups on diagnostic guidelines for Alzheimer's disease. Alzheimers Dement. 2011;7(3):270-279. 21514249 10.1016/j.jalz.2011.03.008 PMC3312027

[17] Palmqvist S, Zetterberg H, Blennow K, et al. Accuracy of brain amyloid detection in clinical practice using cerebrospinal fluid beta-amyloid 42: a cross-validation study against amyloid positron emission tomography. JAMA Neurol. 2014;71(10):1282-1289. 25155658 10.1001/jamaneurol.2014.1358

[18] Benaglia T, Chauveau D, Hunter DR, Young D, (2009c). Mixtools: an R package for analyzing finite mixture models. J Stat Softw. 2009;32(6):1-29.

[19] Bertens D, Tijms BM, Scheltens P, Teunissen CE, Visser PJ. Unbiased estimates of cerebrospinal fluid beta-amyloid 1-42 cutoffs in a large memory clinic population. Alzheimers Res Ther. 2017;9(1):8. 28193256 10.1186/s13195-016-0233-7 PMC5307885

[20] Janelidze S, Pannee J, Mikulskis A, et al. Concordance between different amyloid immunoassays and visual amyloid positron emission tomographic assessment. JAMA Neurol. 2017;74(12):1492-1501. 29114726 10.1001/jamaneurol.2017.2814 PMC5822196

[21] Mormino EC, Betensky RA, Hedden T, et al. Synergistic effect of beta-amyloid and neurodegeneration on cognitive decline in clinically normal individuals. JAMA Neurol. 2014;71(11):1379-1385. 25222039 10.1001/jamaneurol.2014.2031 PMC4293023

[22] Palmqvist S, Janelidze S, Stomrud E, et al. Performance of fully automated plasma assays as screening tests for alzheimer disease-related beta-amyloid status. JAMA Neurol. 2019;76(9):1060-1069. 31233127 10.1001/jamaneurol.2019.1632 PMC6593637

[23] Austin PC, Lee DS, Fine JP. Introduction to the analysis of survival data in the presence of competing risks. Circulation. 2016(6);133:601-609. 26858290 10.1161/CIRCULATIONAHA.115.017719 PMC4741409

[24] Gomez-Gomez ME, Zapico SC. Frailty, cognitive decline, neurodegenerative diseases and nutrition interventions. Int J Mol Sci. 2019;20(11):2842. 31212645 10.3390/ijms20112842 PMC6600148

[25] World Health Organization. Global Status Report on Noncommunicable Diseases 2014*.* World Health Organization; 2014.

[26] Akbaraly TN, Singh-Manoux A, Dugravot A, Brunner EJ, Kivimaki M, Sabia S. Association of midlife diet with subsequent risk for dementia. JAMA. 2019;321(10):957-968. 30860560 10.1001/jama.2019.1432 PMC6436698

[27] Dearborn-Tomazos JL, Wu A, Steffen LM, et al. Association of dietary patterns in midlife and cognitive function in later life in US adults without dementia. JAMA Netw Open. 2019;2(12):e1916641. 31800068 10.1001/jamanetworkopen.2019.16641 PMC6902753

[28] Hosking DE, Eramudugolla R, Cherbuin N, Anstey KJ. MIND not Mediterranean diet related to 12-year incidence of cognitive impairment in an Australian longitudinal cohort study. Alzheimers Dement. 2019;15(4):581-589. 30826160 10.1016/j.jalz.2018.12.011

[29] Kirkpatrick SI, Reedy J, Butler EN, et al. Dietary assessment in food environment research: a systematic review. Am J Prev Med. 2014;46(1):94-102. 24355678 10.1016/j.amepre.2013.08.015 PMC4558887

[30] Wagner M, Dartigues J-F, Samieri C, Proust-Lima C. Modeling risk-factor trajectories when measurement tools change sequentially during follow-up in cohort studies: application to dietary habits in prodromal dementia. Am J Epidemiol. 2018;187(4):845-854. 29020158 10.1093/aje/kwx293

[31] Taylor M, K, Sullivan D, K, Swerdlow R, H, et al. A high-glycemic diet is associated with cerebral amyloid burden in cognitively normal older adults. Am J Clin Nutr. 2017;106(6):1463-1470. 29070566 10.3945/ajcn.117.162263 PMC5698843

[32] Merrill DA, Siddarth P, Raji CA, et al. Modifiable risk factors and brain positron emission tomography measures of amyloid and tau in nondemented adults with memory complaints. Am J Geriatr Psychiatry. 2016;24(9):729-737. 27421618 10.1016/j.jagp.2016.05.007 PMC5003686

[33] Bayer-Carter J, L, Green P S, Montine T J, et al. Diet intervention and cerebro fluid biomarkers in amnestic mild cognitive impairment. Arch Neurol. 2011;68(6):743-752. 21670398 10.1001/archneurol.2011.125 PMC3175115

[34] Hill E, Goodwill AM, Gorelik A, Szoeke C. Diet and biomarkers of Alzheimer's disease: a systematic review and meta-analysis. Neurobiol Aging. 2019;76:45-52. 30682676 10.1016/j.neurobiolaging.2018.12.008

[35] McEvoy CT, Hoang T, Sidney S, et al. Dietary patterns during adulthood and cognitive performance in midlife: the CARDIA study. Neurology. 2019;92(14):e1589-e1599. 30842290 10.1212/WNL.0000000000007243 PMC6448450

